# Loop-mediated isothermal amplification assay for rapid diagnosis soybean damping-off disease caused by *Globisporangium intermedium*

**DOI:** 10.3389/fcimb.2025.1750739

**Published:** 2026-01-16

**Authors:** Xiangrong Zheng, Jian Yu, Jinfeng Peng, Xiuwen Qiu, Yaya Sun, Danyang Fu, Yusufjon Gafforov, Ali Chenari Bouket, Jiajia Chen

**Affiliations:** 1 College of Landscape Architecture, Jiangsu Vocational College of Agriculture and Forestry, Zhenjiang, China; 2 Central Asian Center for Development Studies, New Uzbekistan University, Tashkent, Uzbekistan; 3 Department of Botany and Genetics, Faculty of Biology and Ecology, National University of Uzbekistan, Tashkent, Uzbekistan; 4 Faculty of Chemical Technology, Fergana State Technical University, Fergana, Uzbekistan; 5 Faculty of Digital Technologies, Alfraganus University, Tashkent, Uzbekistan; 6 Plant Protection Research Department, East Azarbaijan Agricultural and Natural Resources Research and Education Centre, Agricultural Research, Education and Extension Organization (AREEO), Tabriz, Iran

**Keywords:** *Globisporangium intermedium*, loop-mediated isothermal amplification detection, on-site disease diagnosis, rapid detection, soybean damping-off

## Abstract

Damping-off has caused huge losses in soybean production areas of China. Based on multi-locus phylogenetic analysis and Koch’s Postulates, the causal agent of soybean damping-off was identified as *Globisporangium intermedium*. Given the potential danger of *G. intermedium*, early and precise detection methods are needed for both disease management and prevention. In this study, a LAMP assay utilizing a new target gene *rpb1* was developed and evaluated for the detection of *G. intermedium*. This *rpb1* LAMP assay was found highly specific to *G. intermedium*. All nine tested isolates of *G. intermedium* yielded positive results, whereas 30 non-target isolates belonging to *Globisporangium* spp., *Pythium* spp., *Phytophthora* spp., *Phytopythium* spp., and soil-borne fungi lacked detection. The LAMP method can identify *G. intermedium* at DNA concentrations as low as 10 pg/μL. In terms of on-site disease diagnosis, the LAMP assay could detect *G. intermedium* from artificially inoculated soybean tissues and naturally infested rhizosphere soil collected from fields in soybean production regions. Consequently, this study not only stands as the first record of *G. intermedium* as a new soybean pathogen in China, but also provides an efficient LAMP field detection method that could significantly contribute to the management and prevention of soybean damping-off.

## Introduction

Damping-off, caused by *Pythium*-like species, have gradually become the most destructive diseases of soybean (*Glycine max* (L.) Merr.), especially in locations with clay soils and rainy periods during sowing. Losses are due to the reduction in plant stand leading to replanting which can increase production costs. Over the past decade, several species of *Globisporangium* were reported as causal agents of this disease (Feng et al., 2020, 2019a; Li et al., 2019; Molin et al., 2021a, 2021b). *Globisporangium* is a recently described genus that was segregated from *Pythium* Pringsheim sensu lato (Nguyen et al., 2022; Uzuhashi et al., 2010). The genus *Globisporangium* includes significant soil-borne and wide-host range phytopathogens with a worldwide distribution, including in the crop-growing regions of Australia, East Asia, North America, and Western Europe (Zhang et al., 2023).

Despite the importance of this genus, accurate identification and classification of *Globisporangium* is quite challenging worldwide (Salmaninezhad and Mostowfizadeh-Ghalamfarsa, 2022). In recent years, molecular techniques for the effective detection of phytopathogenic fungi, viruses, and oomycetes have been widely developed (Chen et al., 2013; Deng et al., 2024; Nath et al., 2014; Nowakowska et al., 2017; Nozawa et al., 2024). Two common approaches are conventional and real-time polymerase chain reaction (PCR). Recent studies have shown that internal transcribed spacer (ITS2) and *cytochrome c oxidase I* (*Cox1*) loci are two effective DNA barcodes for identifying *Globisporangium* spp (Chen et al., 2022; Zhang et al., 2023). However, PCR-based techniques require specialized equipment and are difficult to use for resource-limited laboratories or field diagnoses (Patel et al., 2023). To overcome these obstacles, many isothermal DNA amplification methods, such as loop-mediated isothermal amplification (LAMP), have been developed for on-site diagnosis of plant pathogens rapidly (Chen et al., 2025; Dai et al., 2019; Miles et al., 2015).

Since the LAMP method was first developed by Notomi et al. (2000), it has been widely applied for the detection of various pathogens due to its rapidity, simplicity, and practicality (Feng et al., 2019b). The LAMP reaction enables rapid, continuous, and specific amplification at a low cost, which is well suited for field diagnoses using only a simple water bath or heat block. In addition, the amplification products can be observed with naked eye by adding color indicators, such as HNB (Hydroxynaphthol blue), SYBR Green I/fluorescent SYBR Safe, gold nanoparticle aggregation, or lateral flow dipsticks have been successfully employed, highlighting the flexibility of LAMP for point-of-care diagnosis (Ruang-areerate et al., 2022, 2021; Sriworarat et al., 2015; Suwanngam et al., 2025a, 2025b). Moreover, the LAMP reaction is effective even with crude template DNA extractions (Dai et al., 2019; Shen et al., 2017). Hitherto, several LAMP assays have been developed for rapid detection of *Globisporangium* species, including *G. irregulare* (Feng et al., 2015), *G. recalcitrans* (Chen et al., 2025), *G.* sylvaticum (Zhang et al., 2023) and *G.* ultimum (Shen et al., 2017). Nevertheless, no *G. intermedium*-specific LAMP technology has been developed before this study.

The objectives of this study were 1) to identify and evaluate pathogenicity of *Globisporangium* spp. causing soybean damping-off by means of molecular characterization and Koch’s postulates, respectively; 2) to develop a simple LAMP detection method for specific identification of *G. intermedium*; 3) to evaluate its accuracy in detecting *G. intermedium* in artificially inoculated soybean and *G. intermedium-*infested rhizosphere soil samples.

## Materials and methods

### Strain isolation

**Figure 1:**
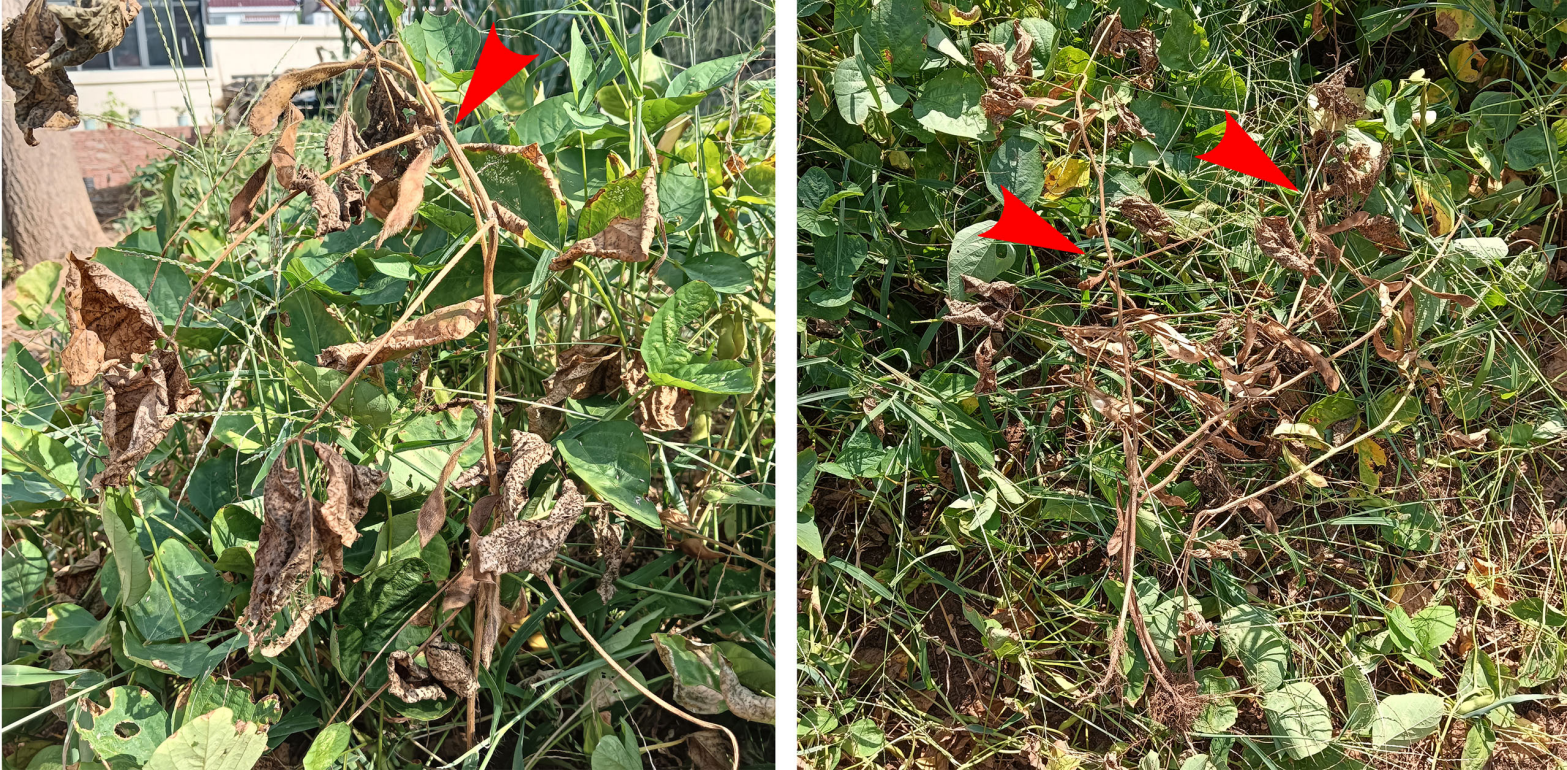
Typical symptoms of damping-off on soybean (*Glycine max*), with the red arrow indicates the diseased soybean.

From 2022 to 2024, soybean samples with typical symptom of damping-off were collected from Jiangsu and Henan provinces of China (**Figure 1**). In total, 56 soybean samples displayed typical symptoms were collected from 11 counties in two provinces of China. The isolates were obtained by incubating the diseased samples at 20°C for 1-2 days in selective V8 juice agar (V8A) medium (15% clarified V8 juice with 2.5 g/L CaCO_3_ and 2% agar) containing rifampicin, ampicillin, and pentachloronitrobenzene and then purifying them using single hyphal or colony tip culture methods. Oomycetes were cultivated on V8A, and the fungal strains isolated for our study were cultured on potato dextrose agar (PDA) medium at 25°C for at least 7 days before DNA extraction. The isolates used in this study are maintained in a collection at Jiangsu Vocational College of Agriculture and Forestry (JSAFC).

### DNA extraction

DNA was extracted from the pure cultures using the Universal Genomic DNA Extraction Kit (Solarbio, Beijing, China) following the manufacturer’s instructions. The ITS and *Cox1* loci were amplified and sequenced with primers ITS5/ITS4 and OomCoxI-Levup/OomCoxI-Levlo, respectively (Robideau et al., 2011; White et al., 1990). PCR amplification and sequencing followed the protocol of Chen et al (Chen et al., 2017). Amplicon sequences were searched for similar sequences in National Center for Biotechnology Information (NCBI) using the basic local alignment search tool (BLASTn).

### Phylogenetic analysis

Phylogenetic reconstruction for the concatenated data set (ITS and *Cox1*) was performed utilizing maximum likelihood (ML) and Bayesian Inference (BI) methods in MEGA 11 (Tamura et al., 2021) and MrBayes 3.2.6 (Ronquist et al., 2012), respectively. *Elongisporangium prolatum* (CBS 845.68) was included as an outgroup. ML tree searches were performed assuming sequences evolved according to the GTR + G + I model and clade support was determined by 1000 bootstrap replicates. For BI analysis, four Markov chains were run for 30 million generations simultaneously, with samples taken from the posterior every 1000 generations. The first 25% of trees were discarded as the burn-in. Bootstrap proportions (BPs) and posterior probabilities (PPs) are used for branch values based on ML and BI, respectively.

### Koch’s postulates

The pure isolates of *Globisporangium* were cultured on V8A at 25°C for three days, and then 8-mm^2^ mycelial plugs were cut from the edge of fresh colonies for pathogenicity tests. According with the inoculation method of Dorrance et al. (2008), hypocotyl slit inoculation technique with minor modifications has been used. Three-day-old soybean seedlings (cultivar Hefeng 47) were selected from germinating surface-sterilized seeds on WA plates. Each soybean hypocotyl was slightly scratched with a sterilized metal needle and placed a mycelial plug in contact with the wound. Each seedling was then transplanted into an individual pot containing 120 g sterilized soil that had been saturated with sterilized water. Each isolate was tested on three seedlings maintained at 25°C with a 12 h photoperiod in greenhouse conditions for 4-5 days. Three seedlings inoculated with sterile V8A plugs served as controls. Each isolate was tested on three seedlings per experimental run, and the entire experiment was independently replicated three times (total n=9 per isolate). After 4 days of inoculation, symptoms of infected seedlings were recorded. Disease severity index (DSI) was evaluated using the scale developed by Türkkan et al. (2022). Differences in severity among isolates was evaluated by the Tukey’s honestly significant difference test (*P* = 0.05) in SPSS 26.0 software (SPSS, Inc., Chicago, IL, USA). To fulfill Koch’s postulates, the infected seedlings were harvested for strain re-isolation on V8 agar medium, followed by ITS sequence comparison with original isolates.

### LAMP primer design and screening

The *rpb1* (RNA Polymerase II Subunit B1) gene was selected as a candidate target for designing *G. intermedium*-specific LAMP primers based on bioinformatics analysis of the gene sequence among *Globisporangium* species. A total of ten LAMP primer sets were designed for *G. intermedium* using Primer Explorer ver. 5 (https://primerexplorer.eiken.co.jp/lampv5e/index.html), followed by a series of tests to assess both specificity and sensitivity (Feng et al., 2019c). Ultimately, an appropriate set was chosen for implementation in this investigation (**Table 1**). A forward inner primer (FIP) consisted of the complementary sequence of F1c and F2, a backward inner primer (BIP) consisted of B1c and B2, two outer primers (F3 and B3), and loop primer (LB) were used for LAMP, and the structure of the universal primers and their complementarity to target DNA is illustrated in **Supplementary Figure S1**.

**Table 1:** Primers used in the *rpb1* loop-mediated isothermal amplification assay.

Primers	Sequences (5’→3’)	Length (nt)^a^
F3 (forward outer)	GAGTTGGGCAGCAACTCG	18
B3 (backward outer)	CCAGCAACAGGCTCTTCG	18
FIP (forward inner) (F1c+F2)	CAGATCCATCGGCAGCAGGT~CTGTTTGCAAAGCGCATTGG	40
BIP (backward inner) (B1c+B2)	CTCGCAGTGACGGCAGCATC~CCGTCCAGTTGATCTTCCTC	40
LB (loop backward)	CGAACAAGCGCATTGTGTCGA	21

a nt, nucleotide.

### Optimization of the LAMP reaction

The LAMP reaction was conducted in accordance with previously described methods (Chen et al., 2025; Dai et al., 2019; Feng et al., 2019a; Zhang et al., 2023). LAMP reaction mixtures with prereaction addition of Hydroxynaphthol blue (HNB) were optimized in preliminary tests by adjusting the concentration of reagents and incubation duration. The final LAMP reaction (26 μL volume) was performed by combining 1 μL *Bst* DNA polymerase (8 U·μL^-1^, New England BioLabs, USA, Cat# M0275L), 3.5 μL dNTPs (10mM, TaKaRa Bio, Japan, Cat# 4030), 4 μL MgSO_4_ (50 mM, Sigma-Aldrich, USA, Cat# M3409), 2.5 μL 10×ThermoPol Buffer (New England BioLabs, USA), 2 μL each of forward inner primer (FIP) and backward inner primer (BIP) (20 μM), 0.5 μL each of primers B3 and F3 (10 μM), 2 μL LB primers (20 μM), 4 μL betaine (5M, Sigma-Aldrich, USA, Cat# B0300), 2 μL HNB (2.4mM, Aladdin, USA, Cat# H196967), 2 μL DNA template, and sterilized double distilled water up to 26 μL. Each set of reactions included a control known to yield a positive detection result (100 ng of purified gDNA of *G. intermedium* as template) and a non-template control (NTC, DNA template replaced by double distilled water). The LAMP reaction mixtures were heated at a range of reaction temperatures (viz., 61, 62, 63, 64, and 65°C) for 63 min to select the optimal temperature (**Supplementary Figure S2**). Additionally, LAMP reactions were performed at the optimal reaction temperature (63°C) for 57, 60, 63, 66, 69 and 72 min to select the shortest viable reaction time (**Supplementary Figure S2**). The assays were evaluated by observation of the HNB color change from violet to blue, which denotes positive amplification, while a negative assay remains violet.

### LAMP specificity and sensitivity test.

**Figure 2:**
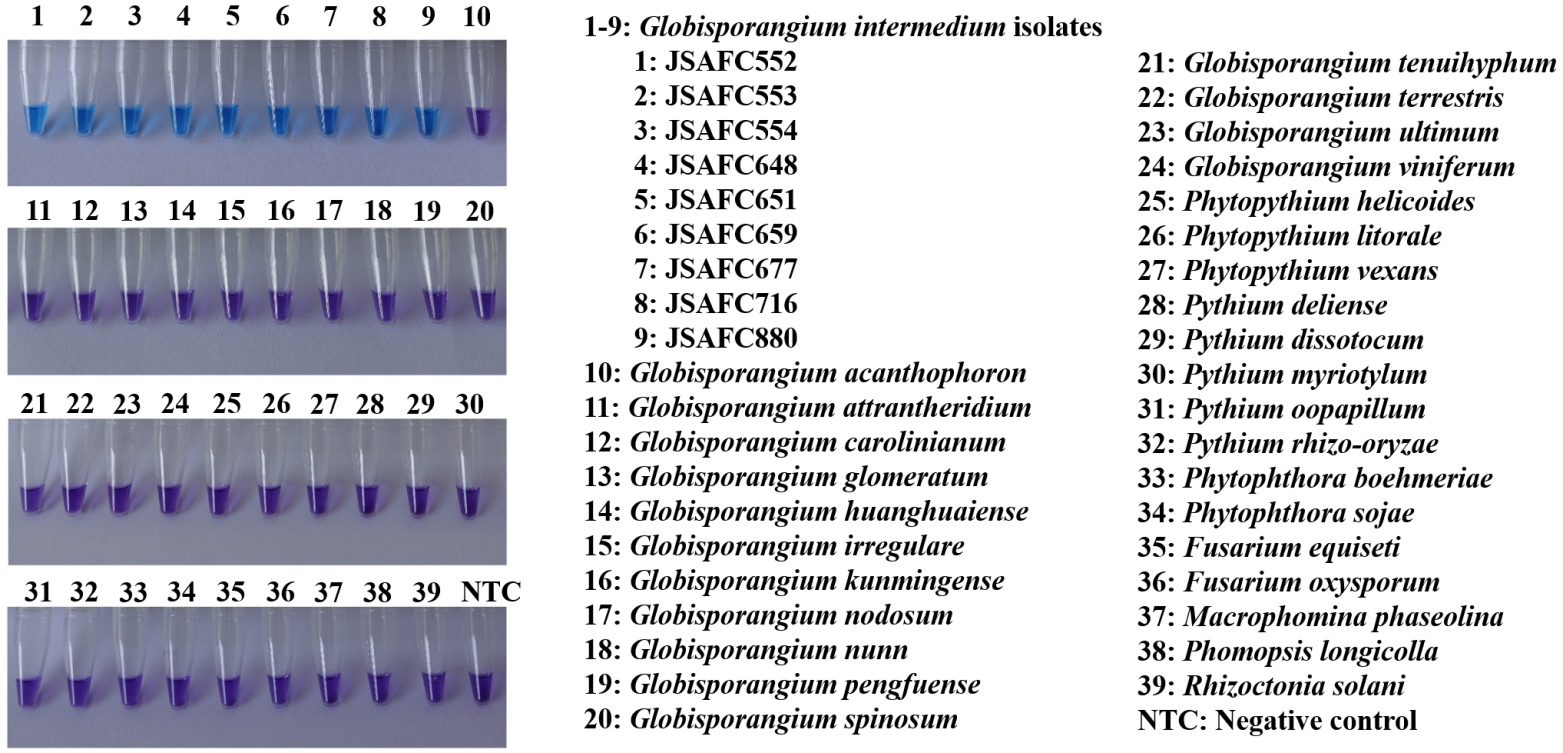
Specificity of the *rpb1* LAMP assay to *G. intermedium*. Blue color indicates the detection of *rpb1* specific to *G. intermedium*. Violet color indicates the lack of detection.

All primer sets were first checked for specificity using 100 ng of genomic DNA template from *G. intermedium* isolate JSAFC651. The isolate JSAFC651 was selected for sensitivity tests due to its robust growth and to challenge the assay under a potentially stringent condition. The established assay conditions were subsequently confirmed to be equally effective for all other *G. intermedium* isolates (**Figure 2**). One LAMP primer set with preliminarily determined specificity was further examined in a wide range of species. In total, 30 isolates of 15 *Globisporangium* species, three *Phytopythium* species, five *Pythium* species, two *Phytophthora* isolates, and five soil-borne fungal isolates were studied (**Table 2**). To evaluate the sensitivity of the method, a series of 10-fold dilutions from 10 ng/μL to 10 fg/μL of *G. intermedium* (JSAFC651) genomic DNA were utilized as templates, with NTC as the negative control. The LAMP assay was performed as previously described with the optimal LAMP primers and reaction parameters. The whole experiment was repeated twice to ensure reproducibility.

**Table 2:** List of oomycete and fungal cultures used in this study, and results of the LAMP assay.

Species	Host(s) isolated	Origin	No. of strains	Result
*Globisporangium intermedium*	Soybean	Jiangsu	4	+
*G. intermedium*	Soybean	Henan	5	+
*G. acanthophoron*	unknown	Hainan	1	**-**
*G. attrantheridium*	Soil	Guangxi	1	**-**
*G. carolinianum*	Soil	Henan	1	**-**
*G. glomeratum*	Soil	Jiangsu	1	**-**
*G. huanghuaiense*	Soybean	Jiangsu	1	**-**
*G. irregulare*	Soil	Jiangsu	1	**-**
*G. kunmingense*	Soil	Henan	1	**-**
*G. nodosum*	Soil	Jiangsu	1	**-**
*G. nunn*	Soybean	Shandong	1	**-**
*G. pengfuense*	Soybean	Jiangsu	1	**-**
*G.* sp*inosum*	Soybean	Jiangsu	1	**-**
*G. tenuihyphum*	Soybean	Jiangsu	1	**-**
*G. terrestris*	Soybean	Jiangsu	1	**-**
*G. ultimum*	Soybean	Shandong	1	**-**
*G. viniferum*	Unknown	Henan	1	**-**
*Phytopythium helicoides*	Soybean	Jiangsu	1	**-**
*Phy. litorale*	Soybean	Jiangsu	1	**-**
*Phy. vexans*	Soybean	Jiangsu	1	**-**
*Pythium deliense*	Soybean	Jiangsu	1	**-**
*P. dissotocum*	Soybean	Jiangsu	1	**-**
*P. myriotylum*	Soybean	Jiangsu	1	**-**
*P. oopapillum*	Soybean	Jiangsu	1	**-**
*P. rhizo-oryzae*	Soybean	Jiangsu	1	**-**
*Phytophthora boehmeriae*	Unknown	Jiangsu	1	**-**
*Ph. sojae*	Soybean	Jiangsu	1	**-**
*Fusarium equiseti*	Soybean	Jiangsu	1	**-**
*F. oxysporum*	Soybean	Anhui	1	**-**
*Macrophomina phaseolina*	Soybean	Jiangsu	1	**-**
*Phomopsis longicolla*	Soybean	Hubei	1	**-**
*Rhizoctonia solani*	Soybean	Anhui	1	**-**

### Detection of *G. intermedium* in artificially inoculated plant samples

To evaluate the ability of LAMP to detect *G. intermedium* in infected plants, soybean cultivar “Hefeng 47” hypocotyls were inoculated with *G. intermedium* discs as mentioned above. Soybean hypocotyls treated with V8A plugs were served as the control group. Subsequently, the ‘Plant-LAMP’ (P-LAMP) detection methods, developed by Feng et al (Feng et al., 2015), has been utilized to analyses the pathogens in soybean tissues. Rotting or browned tissues were cut into 1 cm long (0.2 cm^2^) segments, collected in a 1.5-mL tube, mixed with 100 μL of sterile distilled water, vortexed for 60 s, and then 2 μL of the supernatant was used directly for LAMP amplification. In addition, the hypocotyl samples were placed on V8A and cultivated for 1-3 days at 25°C. The isolates for identification based on their ITS sequences. This experiment was carried out twice. Purified gDNA (100 ng) of *G. intermedium* isolate JSAFC651 was used as the template in a positive control reaction, while NTC served as the negative control.

### LAMP assay using *G. intermedium*-infested rhizosphere soil

Rhizosphere soil samples were collected from locations in Jiangsu and Henan provinces in China from 2022 to 2024, where *G. intermedium* was isolated from soybean roots, during preliminary surveys. A soil sample collected from the *G. intermedium*-free site at JSAFC was used as an additional negative control sample. All samples were transferred to JSAFC and stored at 4°C before further processing. The extraction of DNA from soil samples was efficiently accomplished utilizing a specialized kit known as the FastDNA Spin Kit for Soil (Solarbio, Beijing, China). The experiment was performed along with gDNA of *G. intermedium* (100 ng/μL) and NTC as positive and negative controls, respectively. The experiment was carried out twice.

## Results

### Isolation and identification of *G. intermedium*

The diseased samples were cultured in a selective medium, and 39 *Pythium-*like isolates were obtained. All isolates were initially identified via ITS sequencing, of which 23 showed at least 99.5% similarity to *G. intermedium* type specimen CBS 266.38. Subsequently, 9 of the 23 isolates were obtained from different area and further identified by polygenic phylogenetic analysis.

**Figure 3:**
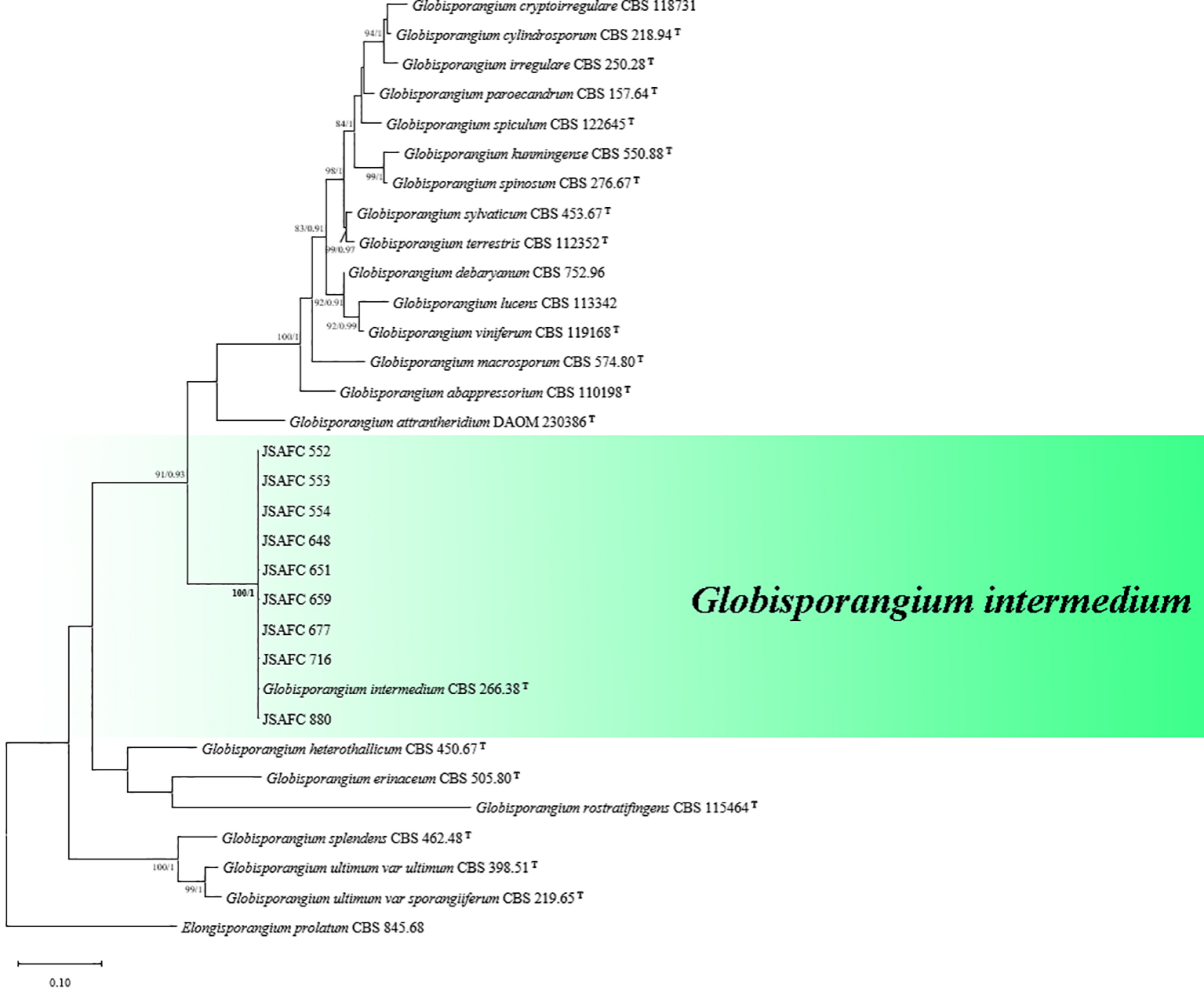
Phylogenetic tree of *Globisporangium* species inferred from a concatenated alignment of ITS and Cox1 sequences. ML bootstrap proportion (BP ≥ 75%) and Bayesian posterior probability (PP ≥ 0.90) were shown at the nodes (BP/PP). Superscript ^T^ indicates the type specimen. *Elongisporangium prolatum* (CBS 845.68) was included as an outgroup. The scale bar indicates the average number of substitutions per site.

The analysis of the combined dataset (ITS + *Cox1*) of our isolates compared to reference sequences from ex-type or other authoritative specimens allowed us to allocate these isolates to molecular groups of known *Globisporangium* species (**Table 3**). The topological structure of the phylogenetic trees constructed using BI and ML criteria was basically consistent, demonstrating that the evolutionary relationships of the experimental strains were statistically supported. A consensus tree with clade support from BP and PP values was generated (**Figure 3**). The multigene phylogeny revealed that all nine *Globisporangium* isolates composed a strongly supported monophyletic clade (100% BP/1.00 PP) with the *G. intermedium* type specimen CBS 266.38, and were clearly distinguished from the other *Globisporangium* species. The nine *G. intermedium* isolates selected for phylogenetic analysis and assay validation originated from geographically distant locations (Jiangsu and Henan provinces) and exhibited unique haplotypes in the concatenated ITS-*Cox1* sequence alignment, supporting their genetic distinctiveness and representative of the pathogen’s population in the surveyed regions.

**Table 3:** A list of species, isolates, and GenBank accession numbers of sequences used in this study.

Species	Isolates^a^	Locality	GenBank Accession No.^b^	
			ITS	*Cox1*
*Globisporangium abappressorium*	CBS 110198^*^	USA	HQ643408	HQ708455
*G. attrantheridium*	DAOM 230386^*^	Unknown	HQ643476	HQ708523
*G. cryptoirregulare*	CBS 118731	USA	HQ643515	GU071825
*G. cylindrosporum*	CBS 218.94^*^	Germany	HQ643516	HQ708562
*G. debaryanum*	CBS 752.96	UK	HQ643519	HQ708565
*G. erinaceum*	CBS 505.80^*^	New Zealand	HQ643534	HQ708578
*G. heterothallicum*	CBS 450.67^*^	Canada	HQ643553	HQ708597
*G. intermedium*	CBS 266.38^*^	The Netherlands	HQ643572	HQ708616
	JSAFC552	Jiangsu, China	*PX069125*	*PX095143*
	JSAFC553	Henan, China	*PX069126*	*PX095144*
	JSAFC554	Henan, China	*PX069127*	*PX095145*
	JSAFC648	Jiangsu, China	*PX069128*	*PX095146*
	JSAFC651	Henan, China	*PX069129*	*PX095147*
	JSAFC659	Henan, China	*PX069130*	*PX095148*
	JSAFC677	Jiangsu, China	*PX069131*	*PX095149*
	JSAFC716	Henan, China	*PX069132*	*PX095150*
	JSAFC880	Jiangsu, China	*PX069133*	*PX095151*
*G. irregulare*	CBS 250.28^*^	The Netherlands	HQ643596	HQ708640
*G. kunmingense*	CBS 550.88^*^	China	HQ643672	HQ708716
*G. lucens*	CBS 113342	Unknown	HQ643681	HQ708725
*G. macrosporum*	CBS 574.80^*^	The Netherlands	HQ643684	HQ708728
*G. paroecandrum*	CBS 157.64^*^	Australia	HQ643731	HQ708772
*G. rostratifingens*	CBS 115464^*^	USA	HQ643761	HQ708802
*G.* sp*iculum*	CBS 122645^*^	France	HQ643790	HQ708831
*G.* sp*inosum*	CBS 276.67^*^	The Netherlands	HQ643793	HQ708834
*G.* sp*lendens*	CBS 462.48^*^	USA	HQ643795	HQ708836
*G. sylvaticum*	CBS 453.67^*^	USA	HQ643845	HQ708886
*G. terrestris*	CBS 112352^*^	France	HQ643857	HQ708898
*G. ultimum* var.*sporangiiferum*	CBS 219.65^*^	USA	HQ643879	HQ708920
*G. ultimum* var. *ultimum*	CBS 398.51^*^	The Netherlands	HQ643865	HQ708906
*G. viniferum*	CBS 119168^*^	France	HQ643956	HQ708997
*Elongisporangium prolatum*	CBS 845.68	USA	HQ643754	HQ708795

a Isolate numbers with an asterisk represent ex-type or other authentic specimens.

b Sequences in italics were generated in this study.

### Pathogenicity test of *G. intermedium*

**Figure 4:**
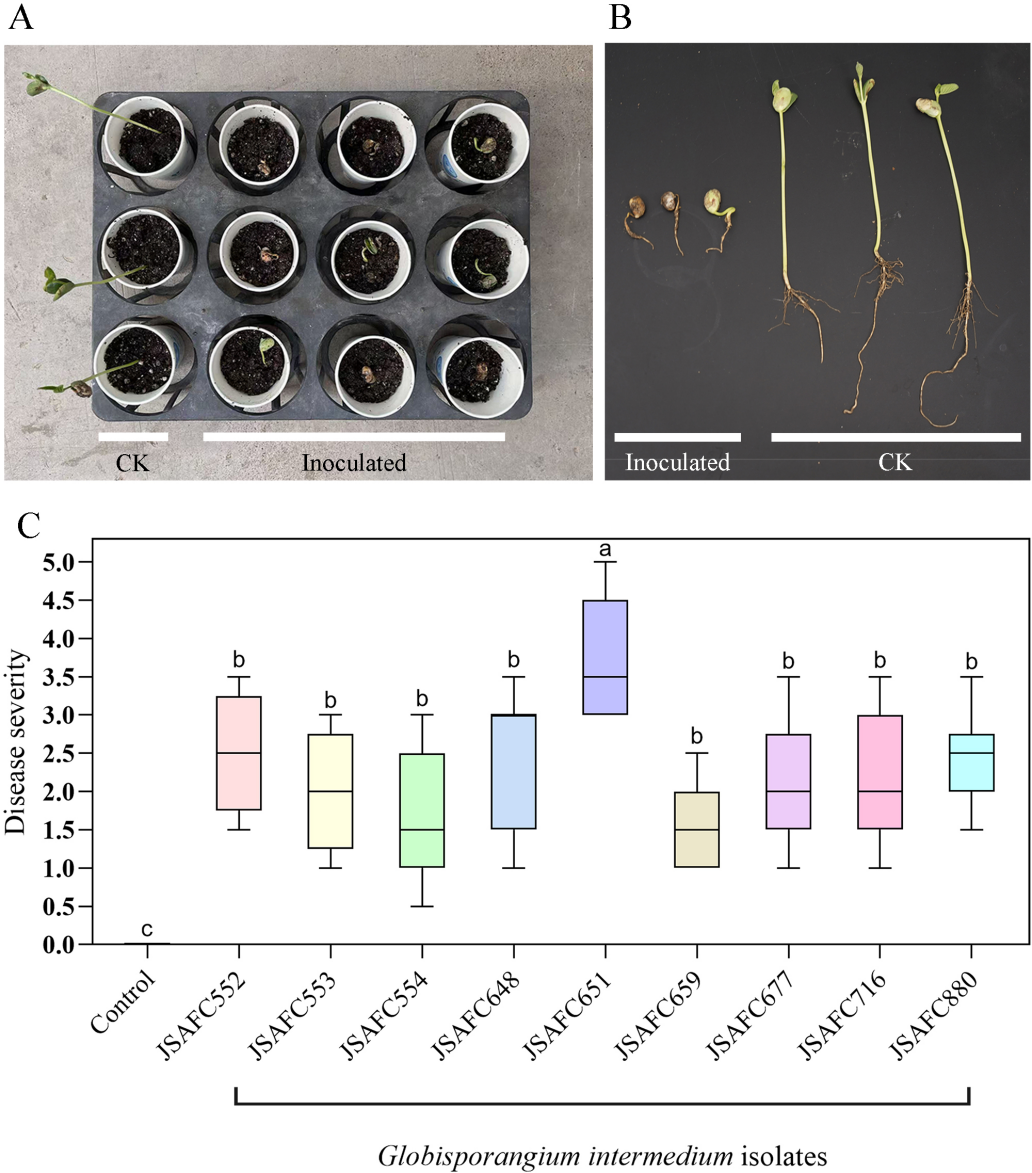
Pathogenicity of *G. intermedium* isolates. **(A, B)** Examples of symptoms observed in the pathogenicity test assay in the non-inoculated (CK) and inoculated soybean seedlings. **(C)** Mean disease severity levels in the pathogenicity test assay. Disease levels were classified as follows: 0 = seedling well developed, no visible symptoms on roots; 1 = seedling well developed, less than 25% of the root system necrotic; 2= seedling growth delayed, up to 50% of the root system necrotic; 3 = seedling growth hampered, up to 75% of the root system necrotic, 4 = death of seedling, complete necrosis of the roots; and 5 = seedlings killed within < one week. Values followed by the same letter are not significantly different according to Tukey’s honestly significant difference test (*P* = 0.05).

The nine isolates selected from the polygenic phylogenetic analysis were further assessed for their pathogenicity. The result indicated that all *G. intermedium* isolates are pathogenic to soybean seedlings (**Figure 4**). When results of the mean disease severity were combined, *G. intermedium* isolate JSAFC651 exhibited higher virulence (mean DSl = 3.78) on soybean seedlings (*P* < 0.05). After four days of inoculation, soybean seedlings were dead after germination, or produced weaker seedlings with significantly shorter plant height, and shorter and fewer lateral roots, whereas those treated with sterile V8A plugs did not display any visible disease symptoms (**Figure 4**). In addition, *G. intermedium* could be reisolated from the symptomatic roots and was verified to be the original isolate based on comparisons of ITS sequences, thus fulfilling Koch’s postulates. These findings validated that *G. intermedium* is a soybean pathogen.

### Development of the LAMP assay for *G. intermedium*

A total of ten primer sets targeting the *rpb1* gene were designed according to the principles of species-specific LAMP primer design, of which showed the greatest specificity were further screened using a broad range of species (**Table 2**). The LAMP reaction mixtures containing gDNAs of nine *G. intermedium* isolates collected from Jiangsu and Henan provinces (**Table 2**) yielded positive detection as the color turned to blue (**Figure 2**). In contrast, LAMP reaction mixtures containing gDNAs of other oomycete or fungal species as well as NTC remained violet in color (**Figure 2**), indicating lack of detection of the target sequence. Two repeated LAMP assays yielded identical results against all tested 39 isolates. This result indicated that the primer set can specifically detect *G. intermedium* isolates. We further optimized the LAMP reactions for the primer set using temperature gradients from 61 to 65°C and reaction time gradients from 57 to 72 min. Considering the high specificity, reactivity, and stability, optimal temperature and time of 63°C and 63 min were selected respectively (**Supplementary Figure S2**).

### Sensitivity of the LAMP assay *in vitro*

**Figure 5:**
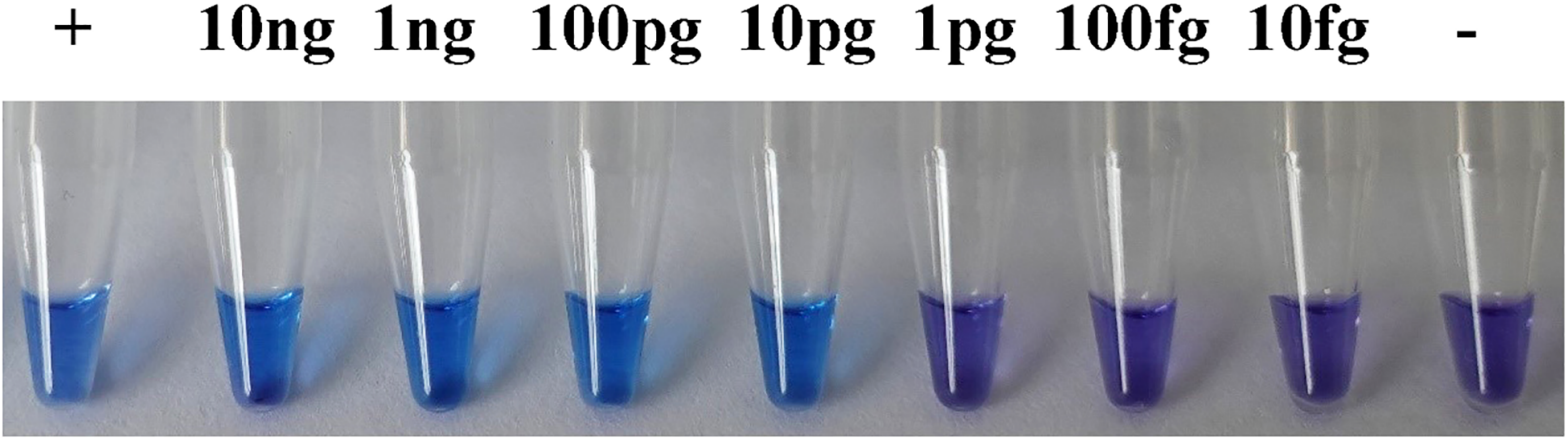
Sensitivity of the LAMP assay to *G. intermedium*. Blue color indicates the detection of *G. intermedium*. Violet color indicates the lack of detection.

Serial dilutions of genomic DNA from *G. intermedium* (JSAFC651) were employed to assess the detection limit of the LAMP method utilizing the chosen primer set at the optimal temperature. The concentrations of the DNA template spanned from 10 ng/μL to 10 fg/μL. As evidenced by a color alteration in the reaction products, the LAMP assay’s lowest detectable limit for *G. intermedium* was 10 pg/μL (**Figure 5**). Identical results were obtained from two replicate experiments.

### Detection of *G. intermedium* in diseased soybean tissues

**Figure 6:**
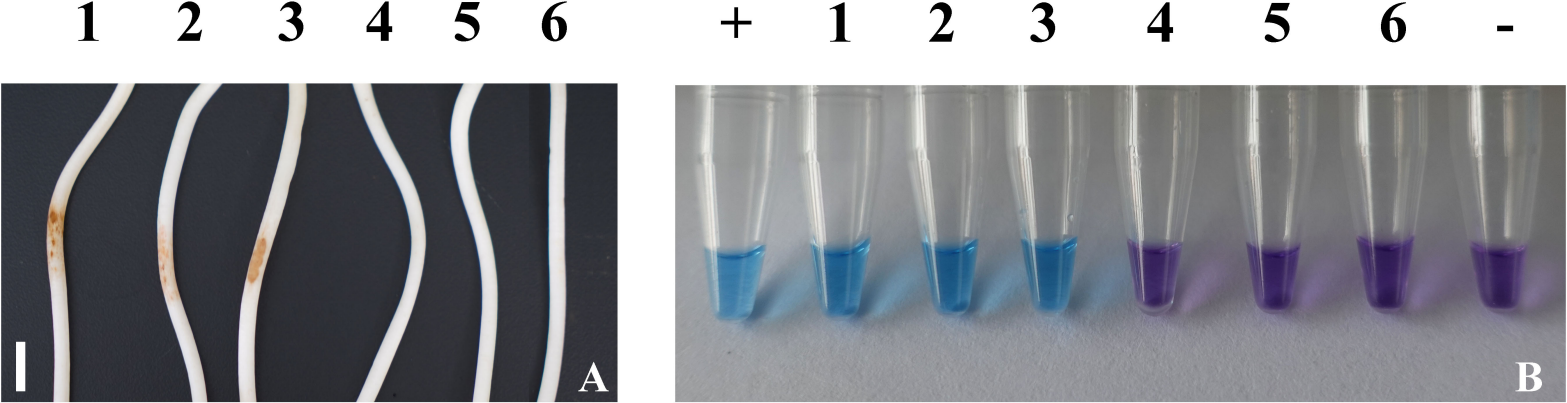
Results of using the LAMP assay to detect *G. intermedium* in inoculated soybean seedlings. **(A)** Diseased (1-3) and healthy (4-6) soybean seedlings, scale bar = 1 cm; **(B)** LAMP results for the detection of *G*. *intermedium* in soybean seedlings. +: Positive control; 1–3: Disease soybean seedlings of inoculate by G. intermedium; 4–6: healthy soybean seedlings; –: Negative control.

Two days after inoculation with *G. intermedium*, typical symptoms emerged at the inoculation sites on the hypocotyls of the soybean cultivar “Hefeng 47”, while no such symptoms appeared on the non-inoculated controls. Crude DNA was then extracted from both the inoculated soybean seedlings and the non-inoculated ones. The LAMP reaction mixtures, incorporating DNA from soybean hypocotyls colonized by isolate JSAFC651, turned blue, paralleling the positive control that utilized *G. intermedium* gDNA as a template (**Figure 6**). In contrast, the non-inoculated samples retained their violet color. These consistent results across two independent assay repetitions highlight the LAMP assay’s high detection capability.

### Detection of *G. intermedium* in naturally infested rhizosphere samples

**Figure 7:**
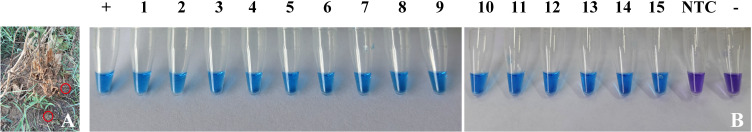
LAMP assay for detection of *G. intermedium* from infested rhizosphere soil in field. **(A)** Diseased soybean plants in the field, with the red circle indicates the sampling area; **(B)** Results of LAMP assay for the detection of *G. intermedium* in infested rhizosphere soil collected in the field. +: Positive control; 1–15: Samples of *G. intermedium*-infested rhizosphere soil; NTC: Negative control, non-template control; –: Negative control, *G. intermedium*-free soil sample.

To evaluate the prevalence of detectable pathogens in field soil samples, we gathered a total of fifteen soil specimens from the vicinity of diseased soybean plants in Henan and Jiangsu provinces, China. When contrasted with the negative control group (one *G. intermedium*-free soil sample and one NTC), the LAMP assay successfully identified *G. intermedium* in the soil of all fifteen rhizosphere samples contaminated with *G. intermedium*, as well as in the positive control (**Figure 7**). The sensitivity, specificity, positive predictive value (PPV), and negative predictive value (NPV) of GNP-LAMP were 100%, 100%, 100% (95% CI, 88.4 – 100.0) and 100% (95% CI, 39.8 – 100.0), respectively (**Supplementary Table S1**). The experiment was replicated twice, yielding consistent results in both runs.

## Discussion

Over the past decade, the cultivation of soybean has undergone a major expansion to meet the increasing demand for bean products in China, which may have caused the high incidence of damping-off in these newly established plantations. Therefore, it is of great importance to diagnose and control this disease. Hitherto, this study stands as9 the inaugural report identifying *G. intermedium* as the causative agent of soybean damping-off in China as well as in the world, offering crucial insights into molecular traits and virulence of *G. intermedium* associated with soybean damping-off.

In this study, *G. intermedium* accounted for 23 out of 39 (59%) *Pythium*-like isolates recovered from diseased soybeans, indicating it is a significant pathogen in the surveyed regions. As a high-risk soil-borne phytopathogen, *G. intermedium* mainly causes damping-off and root rot in various vegetables and crops, leading to severe losses in the agricultural industry (Abdulzahra et al., 2020; Hermansen et al., 2007; Metzger et al., 2007; Rodríguez and Punja, 2007). So far, there has been no research on detecting *G. intermedium* on-site. Thus, an ideal detection assay must be sensitive enough to detect low levels of *G. intermedium* in plants as well as environmental samples, such as rhizosphere samples. Additionally, it must be specific to *G. intermedium*, because misidentification of relatively low-risk *Globisporangium* or other *Pythium*-like species may lead to unnecessary management and removal of plants, which can be costly. In this study, a LAMP assay targeting the *rpb1* gene was developed to detect *G. intermedium* and shown to be specific (**Figure 2**). To the best of our knowledge, this constitutes the inaugural report employing the LAMP assay for the rapid and specific detection of *G. intermedium*.

Target specificity is a cornerstone in the development of molecular detection techniques. Currently, ITS or *Cox1* loci are commonly selected as specific targets for the detection of oomycetes (Feng et al., 2019a, 2019; Wang et al., 2021; Zhang et al., 2023). However, these targets are often insufficient for distinguishing and identifying *G. intermedium* and its closely related species. In this study, through bioinformatic analysis, the *rpb1* gene was ultimately chosen as the detection target. The reaction conditions were subsequently optimized, and detection analyses were performed on nine *G. intermedium* isolates and 30 non-target isolates belonging to *Globisporangium* spp., *Pythium* spp., *Phytophthora* spp., *Phytopythium* spp., and soil-borne fungi strains, culminating in the establishment of a *G. intermedium*-specific LAMP assay. Notably, while the sensitivity of previously reported LAMP assays for other *Globisporangium* species stands at 100 pg/μL of genomic DNA (Feng et al., 2019a), our *G. intermedium*-specific LAMP system attained a remarkable sensitivity of 10 pg/μL. These findings indicated that the developed LAMP assay can accurately detect *G. intermedium* infection in host plants even prior to symptom manifestation.

For on-site detection of *G. intermedium* in field settings, we utilized the P-LAMP method. This approach incorporates a crude DNA extraction technique that eliminates the need for any reagent processing, taking only 1–2 minutes to complete. It has been proven highly effective for detecting *G. sylvaticum* or *Phytophthora colocasiae* in plant roots or taro leaves, as demonstrated in prior studies (Feng et al., 2019b; Zhang et al., 2023). In our study, by testing the diseased hypocotyls of artificially inoculated soybean seedlings, we achieved accurate results (**Figure 6**). These findings confirmed that the samples testing positive for P-LAMP contained *G. intermedium*. Notably, the detection of *G. intermedium* in 15 diseased soybean rhizosphere soil samples further validated the efficacy of this technique in field conditions (**Figure 7**). This efficient and specific detection technology enables the early monitoring and timely warning of *G. intermedium*. Its application not only facilitates timely interventions to minimize economic losses but also supports the development of more scientifically informed control strategies.

While the current HNB-based colorimetric LAMP assay provides a robust tool for the specific and rapid detection of *G. intermedium* in the field, future developments could greatly enhance its utility in complex disease scenarios. To address the common issue of mixed infections causing soybean damping-off, this assay could be integrated with fluorescence-based detection (e.g., using SYBR Safe) to create a dual-indicator system for heightened specificity and the potential for multiplexing. Furthermore, translating this assay onto digital or paper-based analytical devices (dPAD) would enable the absolute quantification of *G. intermedium* DNA, allowing for the assessment of pathogen load or ‘infection density’ in plant or soil samples. Such advancements would move the technology beyond qualitative detection towards a comprehensive, quantitative field-deployable diagnostic system for informed disease management decisions (Saengsawang et al., 2023, 2025; Suwannin et al., 2021).

## Data Availability

The datasets presented in this study can be found in online repositories. The names of the repository/repositories and accession number(s) can be found in the article/**Supplementary Material**.
