# Supplementary material for: Loop-mediated isothermal amplification assay for rapid diagnosis soybean damping-off disease caused by Globisporangium intermedium
Source: Front Cell Infect Microbiol. 2026 Jan 16;15:1750739. doi: 10.3389/fcimb.2025.1750739 (PMC12855517; doi:10.3389/fcimb.2025.1750739)
Supplement: Supplementary file 1 [file DataSheet1.zip › Supplementary Files information.docx]

Figure S1. Design of the LAMP primers specific for *G. intermedium* based on the selected target gene *rpb1*. The location of five core primers [F3, B3, FIP (F1c+F2), BIP (B1c+B2), and LB] are shown. Arrows indicate the 5'->3' direction of primer extension during amplification.

Figure S2. Selection of optimal temperature (A) and time (B) for LAMP assay.

Table S1. Performance evaluation of the *rpb1* LAMP assay for detecting *Globisporangium intermedium*.
