# Supplementary figures and images for: Loop-mediated isothermal amplification assay for rapid diagnosis soybean damping-off disease caused by Globisporangium intermedium
Source: Front Cell Infect Microbiol. 2026 Jan 16;15:1750739. doi: 10.3389/fcimb.2025.1750739 (PMC12855517; doi:10.3389/fcimb.2025.1750739)

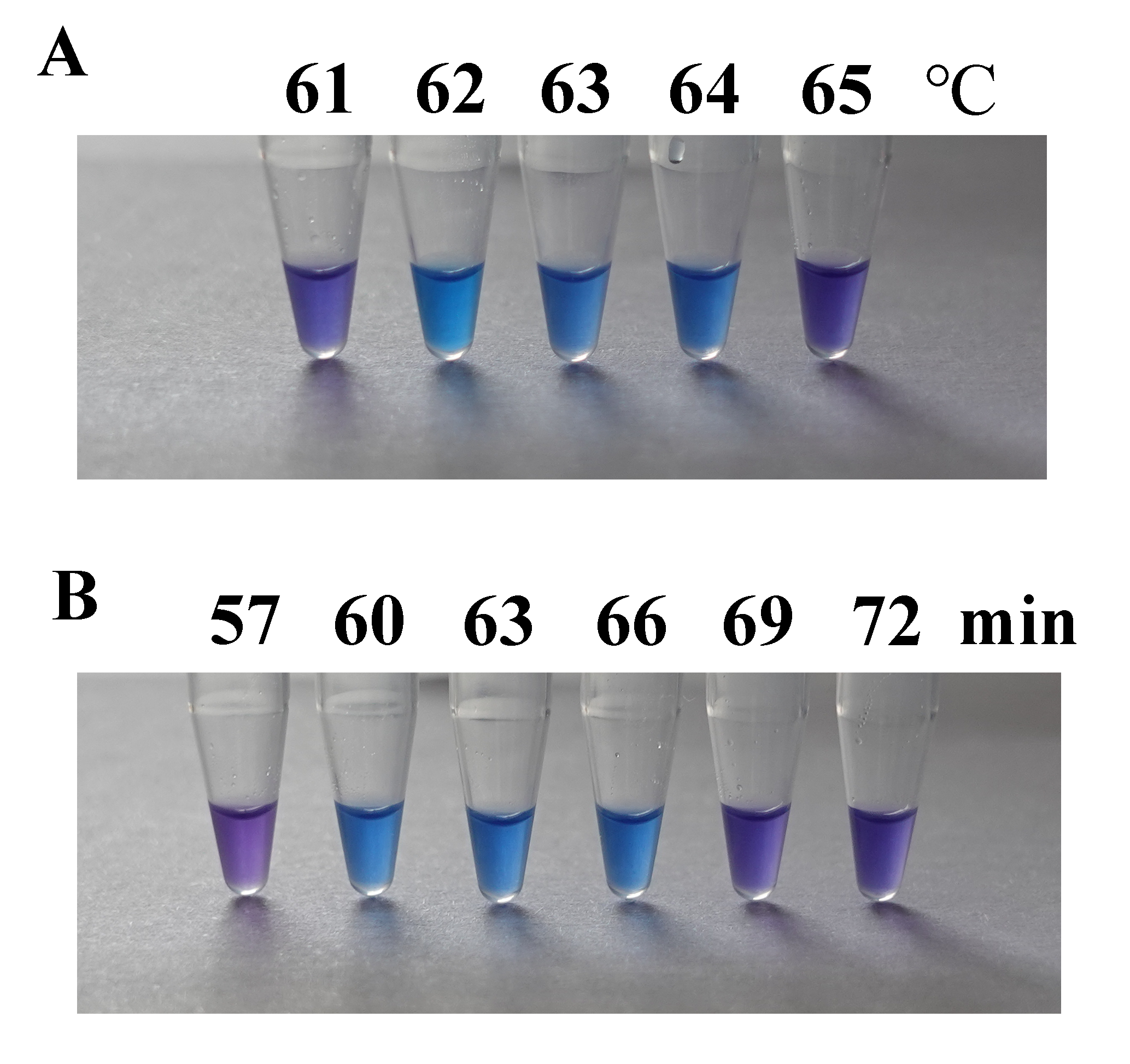

Supplement: Supplementary file 1 [file DataSheet1.zip › Figure S2.tif]

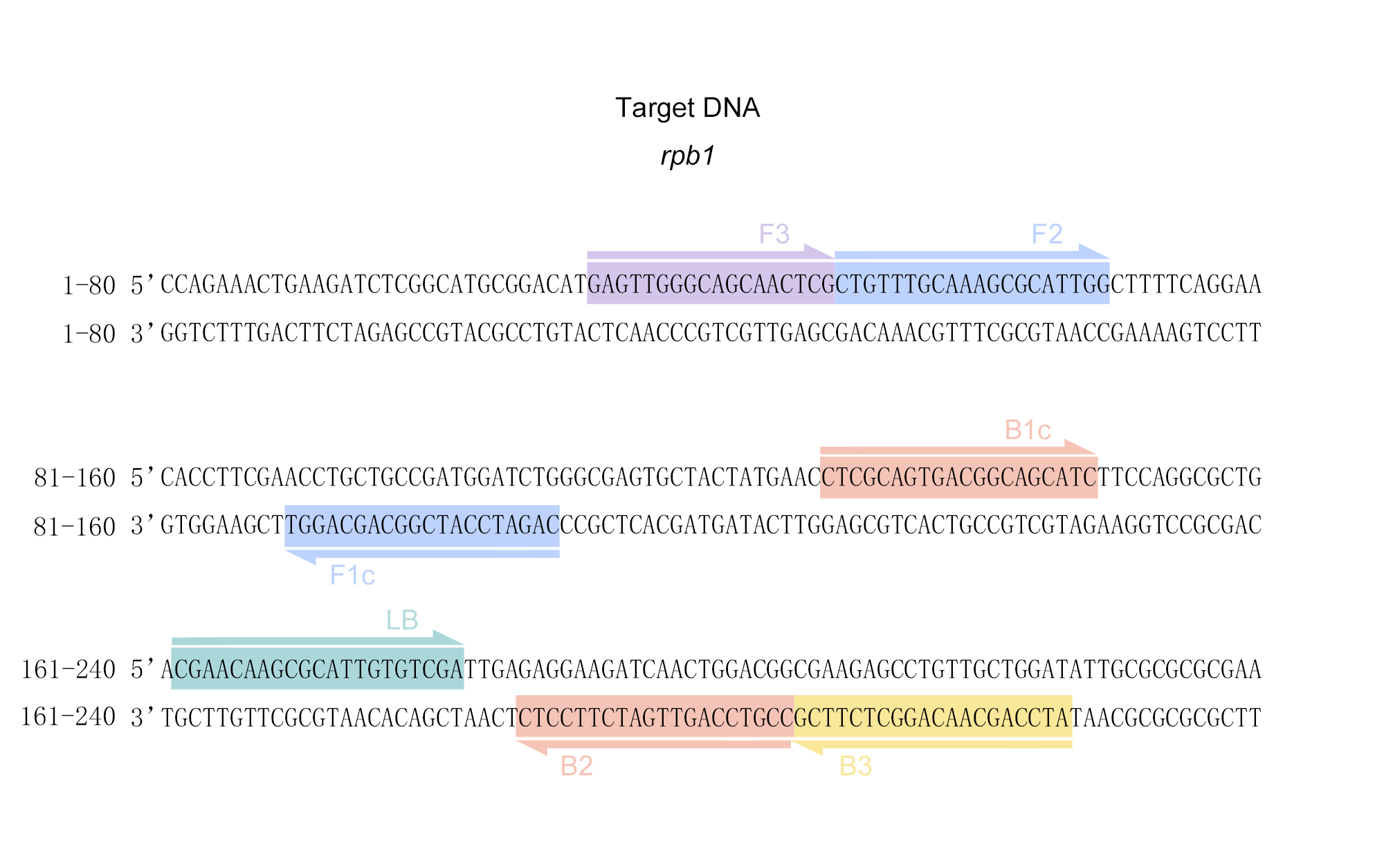

Supplement: Supplementary file 1 [file DataSheet1.zip › Figure S1.tif]
